# Rupture of Rudimentary Horn Pregnancy at 16 Weeks of Gestation

**DOI:** 10.1155/2021/8829053

**Published:** 2021-01-12

**Authors:** Hanane Houmaid, Abderraouf Hilali

**Affiliations:** ^1^Department of Gyneacology and Obstetrics, Mohammed VI Hospital, Chichaoua 41000, Morocco; ^2^Laboratory of Cytogenetics and Toxicogenetics, FST, Settat, Morocco; ^3^High Institut of Health Sciences, Hassan 1st University, Casablanca Road KM 3, 5, PB 539, Settat 26000, Morocco

## Abstract

Pregnancy in the rudimentary horn is rare and a life-threatening. Rupture of pregnant rudimentary horn in the second trimester is a usual presentation. Early diagnosis and fast management are necessary to decrease the mortality and the morbidity of this pathological entity. This report confirms the diagnostic and therapeutic difficulties of the pregnant rudimentary horn. An emergency laparotomy was taken, and ruptured right rudimentary horn was diagnosed. A hemi-hysterectomy was carried out. The patient's postoperative follow-up was uneventful, and she left the hospital 5 days after.

## 1. Introduction

The unicornuate uterus associated with a rudimentary horn is a rare uterine malformation that can lead to several obstetric complications. The incidence of pregnancy in a rudimentary horn is 1/76,000 [1]. The rudimentary horn only communicates with the unicornuate uterus in 10% of cases [2]. The conception is said to be secondary to a migration of sperm through the abdominal cavity before reaching the fallopian tube [3]. The prognosis for pregnancies on rudimentary horn remains bad; the most serious complication is the rupture of the horn. In 80% of the cases, uterine rupture occurs during the first and second trimester [1]. Before the onset of symptoms, the diagnosis is made only in 8% of the cases [4]. We report the case of a uterine rupture on the right rudimentary horn at 16 weeks of gestation; the iconography is particularly illustrative.

## 2. Case Report

A 32-year-old patient, G3P2, with 2 living children from two spontaneous pregnancies. She had an uneventful vaginal delivery, respectively, at 37 weeks and 39 weeks of gestation. The children had a normal growth. The woman had no pathological obstetric history.

She was admitted in a situation of hemorrhagic shock, discolored conjunctiva with generalized abdominal defense in a pregnancy of 16 weeks.

**Figure 1:**
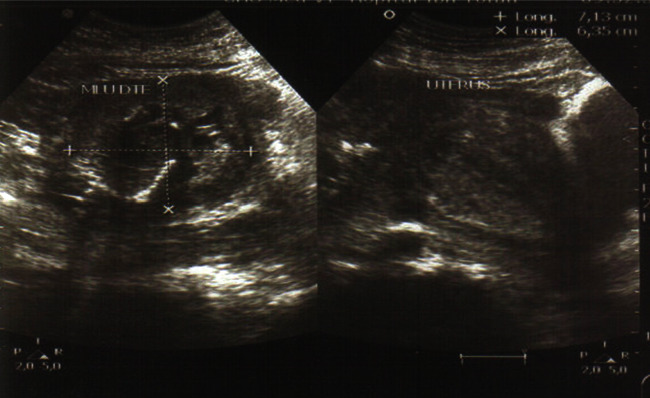
Empty uterus with right uterine lateral mass containing a fetus without cardiac activity.

**Figure 2:**
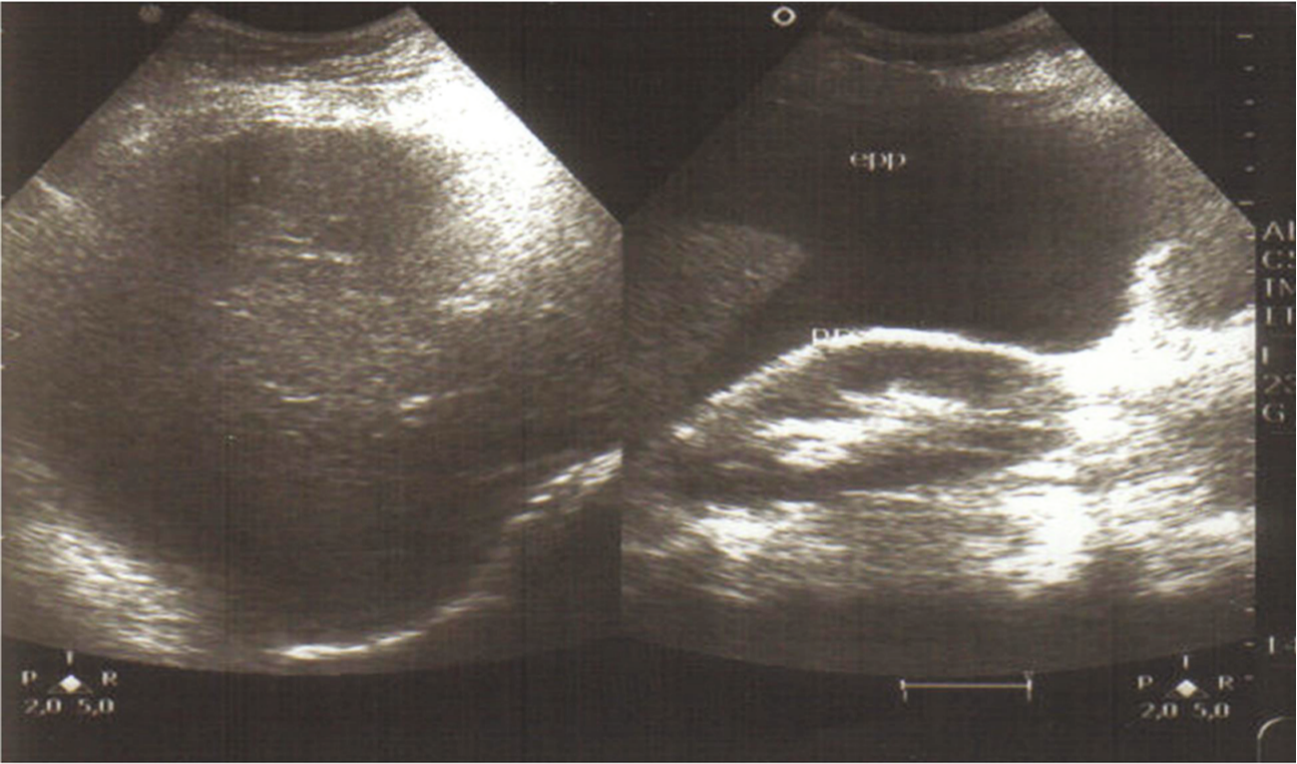
Peritoneal effusion of great abundance.

**Figure 3:**
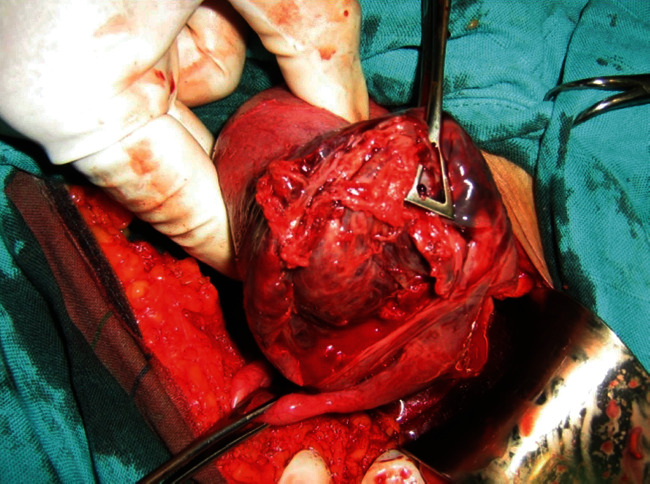
Ruptured rudimentary horn with placenta and fallopian tube ipsilateral.

**Figure 4:**
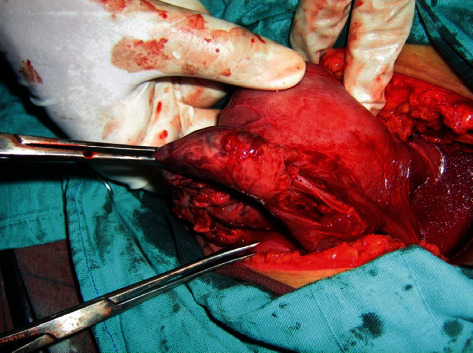
Ruptured rudimentary horn at the right side.

**Figure 5:**
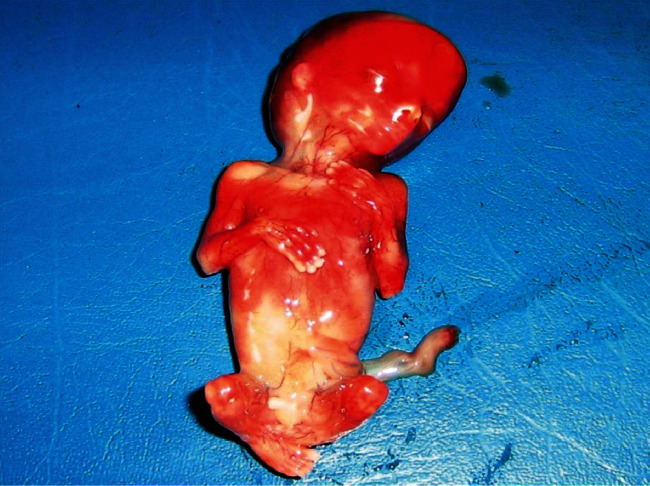
The stillborn fetus was male at 16 weeks of gestation, apparently normal.

The patient also reports the concept of epigastralgia for a week. Pelvic ultrasound showed an empty uterus and an intra-abdominal fetus without cardiac activity (Figure 1) and whose biometry corresponds to a term of 16 weeks with a peritoneal effusion of great abundance (Figure 2). A blood count showed hemoglobin at 4 g/dl. An emergency laparotomy was indicated for suspected ruptured ectopic pregnancy. The exploration showed a 3-liter hemoperitoneum, a fetus externalized intra-abdominally, the rupture of a right rudimentary horn not communicating with the left horn, and the placenta was still attached to the uterus (Figures 3 and 4). The rest of the exploration found normal right and left ovary and fallopian tube with no associated urinary abnormality. We then performed a hemi-hysterectomy with excision of the rudimentary horn and ipsilateral fallopian tube. The stillborn fetus was male, normal in appearance, and weighing 50 grams (Figure 5). The patient received 3 packs of red blood cells and 6 bags of fresh frozen plasma. The postoperative following up was favorable, and the patient left the hospital on day 5.

## 3. Discussion

Uterine malformations, as unicornuate uterus type with rudimentary horn, are due to an abnormality in the development of one of the two Müllerian duct. The unicornuate uterus is class II according to the American Fertility Society's classification of Müllerian anomalies and represents the most common malformation of its kind [5]. When it exists, the rudimentary horn only communicates with the unicornuate uterus in 10% of cases [2], whereas in Musset's classification, there is never any communication between the rudimentary horn and the normal horn. Rudimentary horns are rarely provided with a functional endometrium allowing the implantation of the zygote [6]. The absence of menstrual retention can be explained by the hypoplasia of the endometrium and the aplasia of the isthmus, the presence of which is necessary for the initiation of menstruation. In our case, the horn was on the right side; the embryological tendency to predominate on the right side of the unicornuate uterus remains unexplained. This major uterine malformation is associated in 10% of cases with urinary tract malformations such as agenesis or homolateral kidney ectopia [7] and sometimes bone malformations (spina bifida) or ovarian ectopia. Patients with rudimentary horn are also at higher risk of endometriosis than other uterine malformations [8].

Pregnancy on a rudimentary noncommunicating horn is rare [4]. Just a few cases have been reported in the literature. The fragility and thinness of the rudimentary horn muscle lead to uterine rupture, the most frequent and most serious consequence. This rupture is responsible for an acute hemorrhagic abdominal syndrome similar to a cataclysmic ruptured ectopic pregnancy. It occurs in 90% of cases during the second trimester [9]. A pregnancy in a rudimentary horn could exceptionally lead to a term child if there is an early diagnosis and management [10]. Early diagnosis before rupture is essential to prevent maternal morbidity and mortality. There may be death in utero of the fetus and the formation of a papyraceous fetus.

The prognosis for pregnancy on a rudimentary horn remains dark. Only early diagnosis can prevent uterine rupture on rudimentary horn and improve management in order to reduce maternal mortality and morbidity. A careful abdomino-pelvic examination during the first trimester of pregnancy and the deviation of the normal-sized uterus with latero-uterine mass palpation should suspect a uterine abnormality. This examination is only valuable during the first trimester when the uterus and the rudimentary horn still develop in the pelvis. Some reported cases have been diagnosed by magnetic resonance imaging (MRI) and ultrasound; the sensitivity of which is 26% [4]. This sensitivity decreases with the evolution of pregnancy beyond the first trimester. In our case, the ultrasound, done during the first months of pregnancy, missed the diagnosis which allowed its evolution until the 16 weeks. Although clinical examination or ultrasound may suspect uterine abnormalities, the literature reports a very few cases where the diagnosis is made before the onset of symptoms (8%) and the rate of cases detected preoperatively does not exceed 29% [4].

Confirmation of the diagnosis is often surgical at laparoscopy or laparotomy. Treatment is based on excision of the rudimentary horn and its fallopian tube [11, 12]. A hemi-hysterectomy or total hysterectomy may be necessary to ensure good hemostasis. The incidence of postpartum hemorrhage is high [4], as is the risk of placenta acreta and percreta. Conservative treatment must be reserved for nulliparous women. The interpregnancy interval must be at least one year with good follow-up of the subsequent pregnancy.

## 4. Conclusion

This case is reported to highlight the diagnostic difficulties and to reiterate the need to introduce the possibility of rudimentary horn pregnancy in the differential diagnosis of recurrent abdominal pain during pregnancy. Early diagnosis and fast management reduce the morbidity and mortality of this pathological entity. Treatment is surgical, consisting of resection of the rudimentary horn.
